# Methodology of the French National Study “Context of Sexualities in France” (CSF‐2023)

**DOI:** 10.1111/psrh.70071

**Published:** 2026-08-28

**Authors:** Caroline Moreau, Aline Bohet, Armelle Andro, Yann Le Strat, Nathalie Bajos

**Affiliations:** ^1^ INSERM Soins Primaires et Prevention, CESP Center for Research in Epidemiology and Population Health Villejuif France; ^2^ Department of Population, Family and Reproductive Heal Johns Hopkins University Bloomberg School of Public Health Baltimore Maryland USA; ^3^ Paris 1 Pantheon‐Sorbonne University Paris France; ^4^ Santé Publique Paris France; ^5^ IRIS, Inserm/EHESS/CNRS Aubervilliers France

## Abstract

**Introduction:**

The 2023 national survey “Context of Sexualities in France” (CSF‐2023) explores sexuality and sexual health in France.

**Methods:**

Conducted between November 2022 and December 2023, CSF‐2023 is a cross‐sectional probability survey involving 21,259 respondents aged 15–89 in continental France. Participants were selected via random‐digit dialing, completing a 33‐min phone interview (Part 1), followed by a 30‐min self‐administered online questionnaire for those 18 and older (Part 2). Sexually active respondents aged 18–59 also participated in a sexually transmitted infection (STI) self‐testing study. The survey collected data on sociodemographic characteristics, sexual attitudes, relationships, practices, gender expressions, and digital use for self‐care and sexual practices.

**Results:**

A total of 21,259 individuals completed the Part 1 survey (34% response rate). Of those, 12,906 individuals aged 18–89 completed Part 2 (71% of the 18–89 eligible group). Among the 13,686 individuals aged 18–59 offered STI testing, 4872 (32%) returned results; of the 2959 aged 18–29 offered Human Papillomavirus (HPV) testing, 811 (27%) participated. Post‐stratification weights were applied to mitigate non‐response bias for key sociodemographic traits, with minimal impact on sexual and reproductive health (SRH) indicators, reaffirming sample representativeness. Fieldwork highlighted the importance of not only adapting questionnaire design and sequencing, but also providing strong psychological and supervisory support to interviewers administering a large‐scale survey on sensitive and sometimes distressing topics.

**Conclusion:**

CSF‐2023 provides rich data to track trends in sexuality and sexual health in France, while expanding knowledge on emerging topics such as digital sexual health and SRH self‐care. These findings can inform policy and programming, including the French National Strategy for Sexual Health and the World Health Organization European action plan for SRH. In parallel, the CSF‐2023 fieldwork experience provides important methodological insights into the conduct of large‐scale sexual health research, underscoring the central role of interviewer training, supervision, and psychological support in ensuring high‐quality data collection on sensitive topics.

## Abstract (fr)

**Introduction:**

L'enquête nationale de 2023 “Contexte des sexualités en France” (CSF‐2023) explore la sexualité et la santé sexuelle en France.

**Méthodes:**

Réalisée entre novembre 2022 et décembre 2023, CSF‐2023 est une enquête transversale probabiliste impliquant 21,259 individus âgés de 15 à 89 ans en France hexagonale. Les participants ont été sélectionnés selon une génération aléatoire de numéros téléphoniques. Ils ont rempli un questionnaire téléphonique d'une durée de 33 min (partie 1), suivi d'un questionnaire auto‐administré en ligne de 30 min pour les personnes de 18 ans et plus (partie 2). Les répondants sexuellement actifs âgés de 18 à 59 ans ont également participé à une étude d'auto‐test des Infections Sexuellement transmissibles (IST). L'enquête a recueilli des données sur les caractéristiques sociodémographiques, les attitudes, relations et pratiques sexuelles, les expressions de genre et l'utilisation du numérique pour l'auto‐soin et les pratiques sexuelles.

**Résultats:**

Au total, 21,259 individus ont complété le questionnaire de la partie 1 (taux de réponse de 34%). Parmi eux, 12,906 individus âgés de 18 à 69 ans ont complété la partie 2 (71% du groupe éligible de 18 à 89 ans). Parmi les 13,686 individus âgés de 18 à 59 ans éligibles pour le test des IST, 4872 (32%) ont retourné les résultats; parmi les 2959 âgés de 18 à 29 ans éligibles pour le test du Papillomavirus (HPV), 811 (27%) ont participé. Des pondérations de post‐stratification ont été appliquées pour corriger le biais de non‐réponse en fonction des caractéristiques sociodémographiques clés, sans impact important sur les indicateurs de santé sexuelle et reproductive (SSR), confirmant la représentativité de l'échantillon.

**Conclusion:**

CSF‐2023 fournit des données riches pour suivre les tendances de la sexualité et de la santé sexuelle en France, tout en élargissant les connaissances sur des thématiques émergentes telles que la santé sexuelle numérique et l'auto‐prise en charge en santé sexuelle et reproductive. Ces résultats peuvent éclairer les politiques publiques et les programmes, notamment la Stratégie nationale de santé sexuelle en France et le plan d'action européen de l'Organisation Mondiale de la Sante en matière de santé sexuelle et reproductive. Parallèlement, l'expérience de terrain de CSF‐2023 apporte des enseignements méthodologiques importants pour la conduite d'enquêtes de grande ampleur sur la santé sexuelle, en soulignant le rôle central de la formation des enquêteur·rices, de la supervision et du soutien psychologique pour garantir une collecte de données de haute qualité sur des sujets sensibles.

## 1 Background

According to the World Health Organization (WHO), sexuality is considered “a central aspect of being human”, encompassing a person's identity, attitudes, feelings, and behaviors about intimacy and sexual interactions, and necessitating protection to ensure “pleasurable and safe sexual experiences, free of coercion, discrimination, and violence” [1, p. 5]. Despite the European Union's commitment to these principles, the realization of sexual rights and well‐being in Europe falls short in many respects. Unsafe sex continues to be a measurable source of preventable health loss in Western Europe, including in France, where the burden of disease is slightly above the regional average [2]. Although substantial progress has been made over the past two decades, with mortality and disability‐adjusted life years related to unsafe sex declining by more than 50% since 2004 [2], these gains have been uneven, and disparities by gender, socioeconomic status, race and ethnicity, and sexual orientation persist.

In France, sexual health remains marked by persistent challenges, including sexual violence and discrimination, rising sexually transmitted infections (STIs) among men who have sex with men, and a substantial burden of unintended pregnancy. For example, 14.5% of women report lifetime exposure to sexual violence [3]; STI rates among men who have sex with men have increased [4], and approximately 34% of pregnancies are unintended [5]. Against this backdrop, a thorough analysis of sexual practices and attitudes is essential for informing effective sexual health policies and prevention programs, as well as for evaluating the impact of existing action plans.

Meeting this need requires robust population‐based data. In France, this role has been played by a series of major national sexuality surveys. The Simon survey in 1970 provided an early portrait of sexual behavior in the context of expanding contraception. The ACSF survey in 1992 marked a turning point by placing sexual behavior at the center of public health research in response to the HIV/AIDS epidemic [6]. The 2006 CSF survey was conducted in a transformed context, when HIV was increasingly managed as a chronic condition and issues of gender, inequalities, violence, and sexual rights had become more central [7]. Together with the British National Survey of Sexual Attitudes and Lifestyles (Natsal) programme [8], this body of research holds a distinctive place in the European data landscape [9]. Although other countries have conducted important surveys in this area, few have generated population‐representative data that cover both sexuality and sexual health across such a wide range of topics and over such a long period of time.

Seventeen years after the 2006 CSF survey, the 2023 national survey on sexuality and sexual health, *Contexte des sexualités en France 2023* (CSF‐2023), was launched in a new social era marked by economic and health crises, the digital transformation of intimate life, the legalization of same‐sex marriage and broader recognition of LGBTQ+ rights, renewed public attention to gender‐based violence and sexual consent in the wake of the #MeToo movement, and advances in sexual health prevention and care, including HIV pre‐exposure prophylaxis (PrEP) and expanded access through telemedicine. Commissioned under the French National Strategy on Sexual Health 2017–2030 [10], CSF‐2023 was intended to document and better understand the evolving landscape of sexual practices, relationships, and attitudes in an increasingly diverse society. CSF‐2023 built on earlier French surveys of sexuality and sexual health by extending the age range to 15–89 years, adding an online questionnaire, and incorporating emerging topics, while preserving continuity with prior waves for trend analysis. Taken together, ACSF, CSF, and CSF‐2023 constitute a coherent series of nationwide population‐based surveys that reflect the major social and epidemiological concerns of their respective periods (Table 1).

**TABLE 1:** SRH topics covered in the CSF‐2023 phone survey.

	Part 1	Part 2
Sociodemographics	Gender, age, partnership status, parity, education, employment, country of birth and nationality, parents' education/profession/nationality, race/ethnicity, and religion	Partner Sociodemographics
Sexual education (respondents < 30 years)	SRH topics covered in formal sexual education classes *for minors*	SRH topics covered in formal sexual education classes for 18–29 years
Attitudes toward sexuality	Gender and sexual minorities, gender expectations about sex, importance of sexual activity over the life course	Additional attitudes questions
Sexual orientation and partnerships	Sexual attraction, sexual orientation	Gender identity
Sexual partnerships	Number of male, female, and non‐binary lifetime and 12‐month sexual partners	
Sexual practices	Lifetime and 12 months types of sexual practices and age when first engaged in each practice, period of non‐sexual activity in the last 12 months, number of acts in the last 4 weeks	Details about the first and last sexual intercourse
Digital sexual activity	Partners met online, pornography consumption	Sexual activity online (sexting, pictures, web cameras), pornography consumption
Transactional sex	Providing and consuming	
Sexual violence and discrimination	Type of violence, perpetrator, and disclosing experiences	Gender and sexual discrimination, gynecological violence
Reproductive history	Number of pregnancies, unintended pregnancies, births, abortions, and contraceptive practices	Sexuality during pregnancy and the postpartum period, Infertility, and treatment for infertility
General health	Self‐perceived health, chronic conditions, disability	Mental health (depression PHQ9; anxiety, GAD7) urogenital health
SRH	Sexual dysfunction, sexual satisfaction	Dysmenorrhea Urological/gynecological health
SRH care	Regular medical follow‐up, date of last consultation, STI testing and diagnosis, HPV/hepatitis vaccination, cancer screening (breast, cervical, prostate), sexual satisfaction	Date of last consultation with a healthcare provider, recourse to medical treatment Self‐care practices

This article outlines the protocol of CSF‐2023, detailing the research objectives, hypotheses, survey methodology, and key topics of inquiry.

## 2 CSF‐2023 Objectives and Hypothesis

The CSF‐2023 population‐based study, an interdisciplinary effort, focuses on sexuality and sexual health in France. Its main goals include generating key indicators to track progress toward the 2017–2030 French national strategy on sexual health and exploring trends in sexual attitudes, relations, and practices over time and across geographies (mainland France and overseas territories). Additionally, it examines the impact of the digital revolution on sexuality and sexual health, the realization of sexual rights, and the relationship between sexuality and health.

CSF‐2023 views sexuality as a socially organized practice influenced by various institutions, with norms/attitudes, relationships, and behaviors as its core components. Utilizing an intersectional approach, the study pursues three main lines of inquiry:

### 2.1 Diversification of Attitudes, Practices, and Trajectories

The digital revolution has reshaped social dynamics, creating new boundaries within the sexual landscape. While social media platforms advocate for gender equality and sexual rights, they also present challenges like sexual harassment and conservative movements that threaten the rights of women as well as gender and sexual minorities. These changes likely impact the sexual experiences of young people and contribute to the expansion of online sexual repertoires.

### 2.2 Social Contextualization of Sexuality

Viewing sexuality as a social construct involves examining its context within individual relationships and broader societal frameworks. Increasing social inequalities resulting from economic crises, such as the Great Recession of 2008 and the COVID‐19 pandemic, may influence sexual relationships, particularly for marginalized groups facing challenges related to poverty, autonomy, migration, discrimination, race/ethnicity, or disability. These intersecting conditions, diminishing individuals' sexual autonomy and impeding their ability to lead fulfilling, protected sexual lives free from coercion or discrimination, necessitate further exploration due to their critical impact on sexual health and well‐being.

### 2.3 The Interplay Between Sexuality and Health

While sexual health remains a significant public health concern, technological advancements are improving access to prevention, diagnosis, and treatment options. Therefore, documenting self‐care practices is increasingly necessary to fully encompass the continuum of sexual and reproductive health (SRH) care. Additionally, the connection between sexuality and broader health conditions, including mental health, chronic diseases, and disabilities, underscores the need for a comprehensive, intersectional approach to research, acknowledging variations based on gender, age, and other social positions across the lifespan.

## 3 CSF2023 Methodology

### 3.1 Study Design

The CSF2023 study, conducted in France from November 2022 to December 2023, is a comprehensive, nationwide cross‐sectional probability survey gathering 21,259 questionnaires from individuals aged 15–89 in continental France. The CSF‐2023 comprises a phone questionnaire (Part 1) followed by an online questionnaire (Part 2) alongside a biomarker protocol for STI testing. The study adheres to European General Data Protection Regulation ethical guidelines and received ethical approval from the Committee of Protection of People South East 1 (CPP Sud Est 1, agreement 21.05.19.73038), the Inserm Ethics Evaluation Committee (CEEI‐IRB, agreement 21–868), and the National Commission on Informatics and Liberties (CNIL, agreement DR‐2022‐208).

### 3.2 Pilot Testing and Protocol Refinement

The CSF‐2023 underwent pilot testing in 2020 (interrupted by the pandemic; *n* = 55) and continued in 2021 (*n* = 500), with an additional pilot of the mixed‐mode survey completion design (*n* = 1351).

The pilots led to several important revisions. First, we revised the WHO‐proposed measure of sexual experience [11, 12], defined broadly as any contact with another person perceived as sexual, including kissing, touching, intercourse, or other sexual acts. Respondents often found the term *sexual experience* confusing, and its breadth made comparison with earlier intercourse‐based indicators more difficult. We therefore restructured the survey to begin with age at first sexual intercourse, preserving comparability over time, and then asked about other sexual practices that may have occurred before intercourse. From that point onward, we used the term *sexual practices* to capture a broad range of behaviors, including non‐penetrative forms of sexuality that would not be reflected by the term *sexual intercourse* alone. Second, we shortened the phone questionnaire by 15 min by removing selected items and redistributing content between the phone and online components. We dropped detailed sociodemographic and selected health questions and moved some modules to the online follow‐up. To preserve representativeness, we retained items needed for national estimates in the phone survey, while we shifted questions requiring more in‐depth exploration, such as the context of the last sexual encounter or digital sexual interactions online. We also removed the request for social security numbers for linkage to national health registry claims data in order to reduce respondent burden. Third, we simplified question wording and response formats to improve comprehension and interview flow, including shorter response lists. Fourth, the pilot showed that introducing the STI biomarker sub‐study at the very end of the interview reduced acceptability, likely because of respondent fatigue. In the final survey, we introduced this earlier, alongside questions on STI testing, with a clearer explanation of the home self‐testing procedure and confirmed willingness to participate again at the end of the interview. These changes led to a substantial increase in the STI sub‐study acceptance rates, from 49% in the pilot to nearly 70% in the final survey.

### 3.3 Study Setting and Sample Selection

CSF‐2023 used random‐digit dialing to recruit respondents aged 15 to 89 years living in private households in continental France and able to complete the survey in French. To reduce fieldwork costs, adults aged 65–89 years were undersampled, with only one in two eligible individuals invited to participate.

The target sample size was set at a minimum of 20,000 in continental France, based on estimates from the 2016 French Health Barometer, a national health omnibus survey, to ensure adequate representation of respondents reporting a same‐sex partnership in the past year (2.3% of men and 1.4% of women aged 18–69 years) [13]. Sample size calculations also drew on STI prevalence estimates from the same survey to achieve 1%–2% precision among individuals aged 18–59 years, assuming prevalences of 5% among young women and 3% among young men [14].

Interviewers contacted participants primarily by mobile phone (80%) and 20% by landline, reflecting telephone use in the general population. For landline numbers, the interviewer selected one eligible household respondent from the household roster information; for mobile numbers, the person reached was considered the unique eligible respondent. To improve contact and legitimacy, mobile phone users received an SMS in advance indicating that INSERM would contact them. If there was no answer, the interviewer left a voicemail after the second attempt and sent an SMS after the third attempt; the interviewer also sent an SMS immediately after hang‐up. In total, survey administrators sent 45,682 SMS messages after immediate hang‐ups. This follow‐up helped identify 31% of contacted numbers as ineligible. Among the remaining eligible numbers, 4% resulted in a completed interview.

Interviewers called each number up to 12 times, with up to 8 rings per attempt, with potential extensions during certain periods of low contact rates. Callbacks were scheduled 1.5 to 3 h later, with a maximum of three attempts per day. Numbers were contacted across several days before being dropped, including at least three evening calls and one Saturday call. On average, numbers that resulted in a completed interview were called 8.1 times, and 78% of interviews were completed within 12 attempts.

Initial refusals were recontacted 3 to 5 weeks later by interviewers trained in refusal conversion. This yielded 1956 additional interviews among the 65,890 recontacted, a 3% conversion rate, which accounted for 9% of the final sample (*n* = 21,259). Few refusers agreed to complete the short refusal questionnaire. Still, interviewer feedback and call monitoring suggest that refusals were driven mainly by general survey reluctance rather than the study topic itself.

### 3.4 Interviewer Training and Fieldwork Procedures

Interviewers received study‐specific online 2‐day training delivered jointly by the polling institute's supervisors and the research team. We held five training sessions between October 2022 and June 2023, including a total of 148 interviewers and 16 team leaders. Training covered the study objectives and design, contact procedures, administration of both questionnaire components, the STI biomarker sub‐study, and respondent support procedures. It also included training on the ethical conduct of human subjects' research, including practical guidance on informed consent, confidentiality, sensitive questioning and responding to adult disclosures of a lifetime history of sexual violence. In addition to a detailed questionnaire review, interviewers completed role‐playing exercises and group workshops to prepare for challenging interactions.

### 3.5 Informed Consent Procedures

The interviewers initially provided respondents with detailed information, covering the study's objectives, procedures, risks, benefits, and data rights. Respondents were directed to additional written information on the study website santecsf.fr or received the study leaflet via email, SMS, or regular mail. They could also contact the hotline or visit the study website or Instagram page to verify the study's legitimacy and ask questions. Adult respondents provided oral consent, while parents provided consent for minors 15–17, who then provided assent separately. Consent procedures for STI self‐testing and the Part 2 online survey were conducted at the end of the Part 1 phone interview. Participants were reminded of their data rights in compliance with relevant regulations (Regulation (EU) 2016/679) at the end of the survey.

### 3.6 Data Collection

CSF‐2023 utilized a multimodal data collection approach, starting with the phone survey (Part 1) for all participants and then an online survey (Part 2) for consenting adults. In case of initial refusal of a phone survey, participants were offered to complete Part 1 online, while those who felt uncomfortable responding to Part 2 on the Internet had the option to complete Part 2 on the phone. One hundred thirty‐three trained interviewers conducted phone interviews from November 2022 to December 2023, accumulating 65,000 h of interviews. Interviewers were supported by a supervision team, including one field leader, 14 supervisors, and six listeners, who worked with the research team. This structure supported ongoing quality control through interview monitoring, rapid identification and intervention for field interview challenges, and regular discussion of fieldwork issues. Survey supervisors monitored interviewer‐specific refusal rates throughout the fieldwork. In addition to routine supervision, the research team held debriefing sessions with the interviewers and survey supervisors to review progress, discuss difficulties, and adjust procedures if needed. Weekly or bi‐weekly voluntary sessions with a psychologist were offered to support interviewers in managing sensitive interactions, including disclosures of violence, as well as exposures to sexual harassment or aggressive behavior, and managing stress related to the survey's subject matter. Additional one‐on‐one sessions were also available. Midway through fieldwork, the research team organized four 90‐min refresher debrief sessions to provide an opportunity to take stock of interviewer experiences, discuss mindset and practical points for questionnaire administration, and revisit difficult situations with the psychologist.

### 3.7 Questionnaires

#### 3.7.1 Part 1 Survey

The CSF‐2023 survey was designed to delineate patterns of sexual norms, attitudes, practices, and health for trend analysis and cross‐national comparisons, while also exploring new areas of interest. The first Part, conducted mostly by phone, lasted an average of 33 min (excluding the consent process). The questionnaire incorporated questions from previous French national surveys on sexuality and SRH (1992–2006) [6, 7] and adopted the WHO's standardized questions for sexual behavior [11, 12]. We also introduced new modules to explore gender expressions, sexual experiences at different life stages (pregnancy/postpartum), and digital media use for self‐care and sexual practices (Table 1).

#### 3.7.2 Part 2 Survey

A 30‐min online questionnaire was offered after Part 1 and was accessible via a secure link. Participants were encouraged to complete it within 4 weeks to ensure data synchronization, although the timeframe was expandable. Interviewers sent reminder emails/SMS with secure links on days 3, 6, and 10, with a final phone follow‐up on day 15. The online survey expanded on Part 1 topics and collected additional information on first and last sexual experiences, online sexual activity, last sexual partner, and SRH care‐seeking behaviors. Parts 1 and 2 questionnaires were developed in French and maintained in French throughout the entire study period.

#### 3.7.3 Biomarkers

Participants aged 18–59 who responded to Part 1 by phone and reported ever having sexual intercourse were invited to join the biomarker sub‐study (PrevIST). PrevIST aimed to estimate the prevalence of common bacterial STIs, including *Chlamydia trachomatis, Neisseria gonorrhoeae, and Mycoplasma genitalium* in France. The *Human Papillomavirus Virus* (HPV) was also estimated among adults aged 18 to 29 who had ever had sexual intercourse. Interviewers provided detailed information on the objectives of PrevIST, STI self‐testing procedures, confidentiality, and how to return the results. STI testing kits containing study materials (documentation, consent forms, vaginal swab for women, a urinary container for all men, and a penile swab (HPV testing) for men aged 18 to 29 years) were dispatched within 6 days of the phone survey. Participants returned specimens and consent forms to the National Reference Center for Bacterial STIs (CNR IST) for laboratory testing. Results were sent within 4 weeks. Survey administrators contacted participants who didn't return their kits up to four times, starting 10–30 days after mailing (email on day 12, phone on day 17, and email or SMS on days 22 and 29).

## 4 CSF‐2023 Response Rates

### 4.1 Part 1 Survey

We report study response rates following the American Association for Public Opinion Research (AAPOR) 10th edition [15]. Out of 809,031 randomly generated phone numbers, 315,391 were deemed valid for the study after excluding professional, fax, or false numbers. Eligibility criteria included individuals aged 15–89, with half specifically selected for individuals aged 65–89. Exclusions consisted of 155,777 numbers with no contact, 21,024 non‐eligible numbers (10, 194 outside the age range, 5197 non‐Francophone, 3834 physically or mentally unable to participate, and 1524 absent during the study or not a household member, and 275 secondary residence numbers). While 315,391 numbers were potentially eligible, confirmation was obtained for only 82,665 individuals due to unsuccessful contact attempts (for 138,590 households), unsuccessful recontact after initial contact (35,140 numbers), and immediate refusals (20,785) before eligibility screening. Another 56,355 eligible individuals refused to participate, while 21,259 completed Part 1 (19,150 on the phone and 2109 on the internet), and 5051 partially completed the questionnaire.

Using the APOOR outcome rate calculator for random‐digit‐dial surveys, we estimated a response rate of 34%, a refusal rate of 28%, and a cooperation rate of 40% based on completed questionnaires. The cooperation rate rose to 60% if we included incomplete questionnaires. Response rates were similar by phone type (cell vs. landline), but cooperation and refusal rates differed, with greater cooperation and lower refusal among cell phone users than landline users. A summary of APOOR disposition codes, along with the response rates, refusal, and cooperation rates calculations are reported in Table 2.

**TABLE 2:** Distribution of call outcomes according to APOOR disposition codes and estimates of participation, refusal, and cooperation rates.

	Disposition codes	Total	Landlines	Cell phones
**Interview (category 1)**	**1.00**	**26,310**	**3960**	**22,351**
Complete	1.10	21,259	3296	17,963
Partial	1.20	5051	664	4388
**Eligible, non‐interview (category 2)**	**2.00**	**17,402**	**4094**	**13,308**
Refusal	2.11	16,280	3936	12,344
Break off	2.12	1122	158	964
**Unknown eligibility, non‐interview (category 3)**	**3.00**	**94,878**	**27,866**	**67,012**
Household exists; unknown if the eligible respondent	3.20	35,140	7081	28,059
Screener refused	3.21	260	260	
Other unknown eligibility	3.90	59,478	20,525	38,953
**Not eligible (category 4)**	**4.00**	**176,801**	**98,638**	**78,163**
**Total sample use**		315,391	134,558	180,834
**I = Complete**	1.10	21,259	3296	17,963
***R* = Partial**	1.20	5051	664	4388
***R* = Refusal and break‐off**	2.11 & 2.12	17,402	4094	13,308
**NC = Non contact**	2.2	0	0	0
**O = Other**	2.3 & 2.9	0	0	0
**Coefficient** (*Based % eligible among known eligibility cases*)		0.198	0.075	0.313
**UH = Unknown household**	3.1	0	0	0
**UO = Unknown respondent eligibility**	3.2 & 3.9	94,878	27,866	67,012
**Response rate**		0.340	0.324	0.317
I/((I + *P*) + (*R* + NC + O) + e(UH + UO)) Complete/((complete+partial) + (refusal+Non contact + other) + coef (Unkown househol + Unknown respondent eligibility))				
**Cooperation rate**		0.48	0.409	0.504
I/((I + *P*) + R) Complete/(complete + partial + refusal)				
**Cooperation rate**		0.602	0.409	0.627
Complete + partial/(complete + partial + refusal)				
**Refusal Rate**		0.278	0.403	0.235
R/((I + *P*) + (*R* + NC + O) + e(UH + UO)) Refusal/(complete + partial ++ Non contact + other) + coef (Unkown household + Unknown respondent eligibility)				

### 4.2 CSF‐2023 Sub‐Studies

A total of 21,066 adult participants were eligible for the Part 2 survey extension, with 18,066 accepting (93%), 12,906 completing the most relevant sections of the questionnaire (71%), and 12,615 completing Part 2 in its entirety. Among 15,280 adults aged 18–59 who reported ever having sexual intercourse in Part 1, 13,686 were offered to participate in the STI biomarker sub‐study (those who responded to Part 1 by phone), and 4872 (32% of the eligible sample) returned results that were analyzed. Among 3261 adults aged 18–29 who reported ever having sexual intercourse in Part 1, 2959 were offered to participate in the STI biomarker sub‐study (those who responded to Part 1 by phone) and 811 (27%) accepted and returned results for analysis. Figure 1 summarizes sample selections across sub‐studies.

**FIGURE 1:**
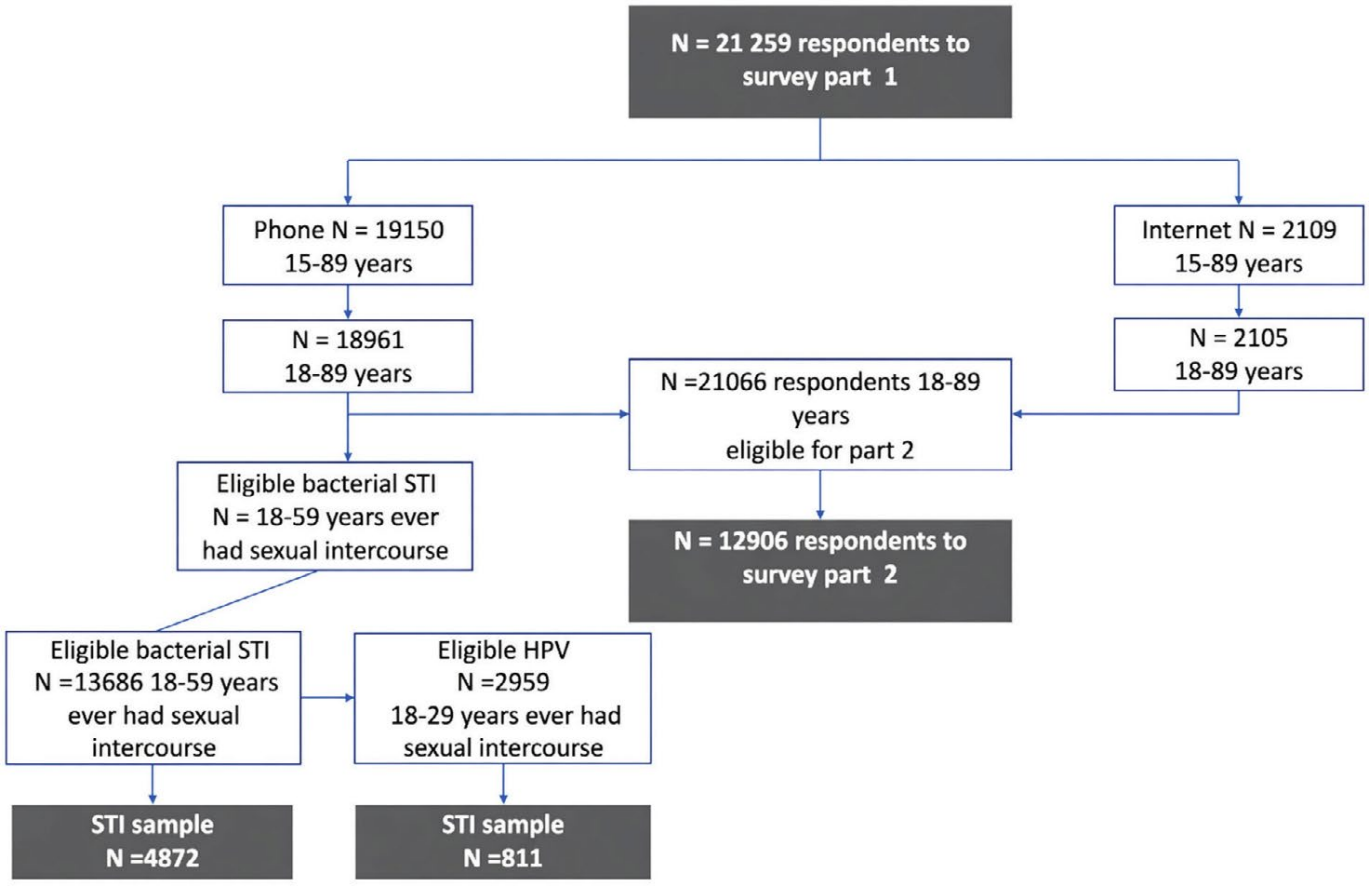
Sample flow chart by data collection mode and sub‐studies.

#### 4.2.1 Non‐Response Bias Analysis and Post‐Stratification Weights

We constructed sample weights to account for the initial probability of selection by age group, and post‐stratification; once we established these base weights, we calculated additional nonresponse/attrition weights separately for the internet sub‐sample (Part 2 internet), the bacterial STI subsample, and the HPV sub‐sample.

##### 4.2.1.1 Non‐Response Bias Analysis and Post‐Stratification of CSF‐2023 Initial Sample (Part 1, Phone Survey Sample)

Table 3 compares the sociodemographic distribution of the Part 1 sample to the national 2023 census distributions. The unweighted Part 1 sample matched the census data for region and household composition (number of household members) but showed distortion by age and educational attainment. By design, the Part 1 sample underrepresented people aged 65 to 89, as only half of the eligible adults aged 65–89 were included to reduce study cost. Additionally, the Part 1 sample underrepresented minors (15–17) who needed additional parental consent to participate. The sample also grossly underrepresented people with less than a high school degree and, conversely, overestimated people with some college or graduate education.

**TABLE 3:** Unweighted and weighted sample distribution compared to census data.

	*N* respondents, part 1 21,259	Census data 2023	Unweighted sample part 1	Weighted sample part 1
Sexe				
Men	10,235	48%	48%	48%
Women	11,019	52%	52%	52%
Other	5		< 0, 1%	< 0,1%
Age				
15–17 years	193	5%	1%	4%
18–19 years	557	3%	3%	3%
20–24 years	1675	7%	9%	7%
25–34 years	3383	14%	16%	14%
35–44 years	4086	15%	17%	16%
45–54 years	4192	16%	17%	17%
55–64 years	4110	16%	16%	16%
65–74 years	1896	14%	14%	13%
75–89 years	1167	11%	8%	10%
Highest diploma				
No diploma	752	13%	4%	11%
Less than high school graduation	4357	37%	22%	37%
High school graduation	3981	18%	19%	19%
Some college (1–2 years)	5833	20%	27%	21%
College graduation or graduate degree	6315	11%	28%	11%
Missing	21		< 0.1%	< 0.1%
Region				
Auvergne Rhone Alpes	2822	12%	13%	13%
Bourgogne Franche Comte	883	4%	4%	4%
Bretagne	1183	5%	6%	5%
Centre Val de Loire	859	4%	4%	4%
Corse	88	1%	1%	1%
Grand Est	1644	9%	8%	9%
Hauts de France	1582	9%	8%	9%
Ile de France	3994	19%	18%	18%
Normandie	997	5%	5%	5%
Nouvelle aquitaine	2097	9%	10%	10%
Occitanie	2114	9%	10%	10%
Pays de la Loire	1369	6%	6%	6%
Provence alpes cote d'Azur	1604	8%	8%	8%
Missing	23		< 0.1%	< 0.1%
Number of household members				
1	4786	20%	23%	21%
2	6982	35%	36%	35%
3	3611	18%	16%	18%
4	3827	16%	16%	16%
5 or more	2053	10%	9%	10%

To account for differential inclusion probabilities and sample distortion, we created sampling weights following the procedures outlined below. Initial sample weights were determined as the inverse probability of selection, accounting for the number of phone lines per participant and age (65–89 years were undersampled). Subsequently, to address non‐response bias, post‐stratification weights were calculated to match the 2023 census data. This involved performing margin calibration in R, crossing age and sex, using region of residence (13 regions), educational attainment, and household size (number of household members). We truncated extreme weights (1% percentile and 99% percentile) using the Taylor Series Weight Trimming Procedure [16] to reduce the max‐min weight ratio from 330 (min = 162, max = 13,595) to 37 (364–13,384) weight ratios.

Post‐stratification weights successfully corrected for sample distortion, reducing all discrepancies to less than 1 percentage point except for no diploma, which remained two percentage points lower in the weighted phone sample than in census data.

Additional analysis of the impact of post‐stratification weights on key sexuality indicators shows little change after applying weights with a 1.2 percentage point increase in the percentage of respondents who indicated an age at first sex of less than 15 years (13.9% unweighted, 15% weighted), a 1.4% decrease in the percentage of individuals who indicated having had same‐sex partners (9.3% unweighted, 7.9% weighted), and a 1 percent difference in the percentage of respondents who reported more than one partner in the last 12 months (11% unweighted, 10.1% weighted).

##### 4.2.1.2 Non‐Response Bias Analysis and Post‐Stratification of CSF Sub‐Study Samples (Part 2 Online Sample, STI Subsample and HPV Sub‐Sample)

To further account for selection bias in each sub‐study sample (Part 2, STI and HPV), we compared the distribution of sociodemographic and sexual health indicators between eligible respondents to each sub‐study and actual sub‐study participants. We found that the Part 2 sample distribution matched the Part 1 eligible sample distribution of respondents aged 18–89 within a 2‐percentage‐point margin for all indicators except education and type of employment. The STI and HPV sub‐sample distributions (for which STI and HPV results were available) differed substantially from those of the eligible STI and HPV Part 1 sample populations (more than 4 percentage points).

To adjust for selective participation in the nested sub‐studies, we estimated separate inverse probability of participation models for each sub‐sample (Part 2, STI, and HPV) using the corresponding eligible Part 1 respondents as the reference population. For Part 2, participation was modeled as a function of sociodemographic factors (age, education, country of birth, children), sexual health indicators (lifetime and 12‐month sexual partners, lifetime same‐sex partnerships), perceived health, and reported difficulty completing Part 1 survey to estimate each participant's probability of inclusion in Part 2. Given the high correlation between education and type of employment and the lack of statistical significance for marital status, residence, and financial situation in multivariate analysis, these factors were not included in the regression analysis. We followed the same strategy separately for the STI and HPV samples, adding completion of Part 2 in the models because it was a strong predictor of participating in PrevIST. For each sub‐sample, the predicted probabilities were converted into inverse probability weights, and these were multiplied by the original Part 1 weight to produce the final analysis weight for that sub‐study. Table 4 presents the distribution of eligible respondents for each sub‐study, along with the unweighted and weighted distributions for each sub‐study sample. Although occupational social class was not included in the weighting models because it was highly correlated with education and only available for employed respondents, we compared its distribution in the eligible samples and weighted sub‐subsamples. The weighted distributions matched eligible samples within margins of 2% for Part 2, 2% for the STI sample (except for a 3% difference for lower and worker professions), and 4% for the HPV sample (except for lower education and profession).

**TABLE 4:** Post‐stratification unweighted and weighted distributions of Part 2, STI and HPV sub‐samples compared to weighted eligible respondents from Part 1.

	Sample part 2 (adults 18–89 years)			Bacterial STI sample adults 18–59 years who ever had intercourse			HPV sample adults 18–59 years who ever had intercourse		
	Eligible	Unweighted	Weighted^a^	Eligible	Unweighted	Weighted^b^	Eligible	Unweighted	Weighted^c^
Total (*N*)	21,066	12,906	12,906	15,280	4872	4872	3261	811	811
Gender									
Men	48%	47%	48%	50%	43%	49%	50%	40%	50%
Women	52%	53%	52%	50%	57%	51%	50%	60%	50%
Age									
18–19	18%	16%	18%	23%	19%	23%	14%	10%	15%
20–24 years							41%	41%	45%
25–29 years							45%	49%	39%
30–39 years	16%	15%	16%	24%	28%	24%			
40–49 years	16%	16%	16%	24%	27%	24%			
50–59 years	18%	19%	18%	28%	31%	29%			
60–69 years	16%	18%	16%						
70–79 years	13%	13%	12%						
80–89 years	4%	3%	4%						
Married	40%	42%	40%	32%	35%	30%	4%	4.2%	3%
Children	70%	71%	70%	64%	66%	63%	13%	10%	12%
Education									
< High school	47%	40%	46%	38%	27%	41%	25%	13%	31%
High school	20%	21%	20%	22%	20%	20%	37%	35%	32%
Bachelor	22%	25%	22%	26%	31%	26%	26%	32%	25%
Graduate	12%	14%	12%	14%	21%	13%	12%	19%	11%
Financial difficulty	40%	38%	40%	43%	37%	45%	42%	39%	46%
Born in France	90%	93%	90%	89%	93%	90%	90%	94%	90%
Residence									
Large city	27%	27%	27%	30%	32%	29%	39%	47%	41%
Medium size city	38%	37%	38%	37%	34%	35%	38%	34%	35%
Rural	35%	35%	35%	34%	34%	36%	23%	19%	24%
Number of lifetime same‐sex partner	8	9	8	10	13	10	14	19	12
Sexual partners last year									
0	29%	27%	29%	16%	12%	17%	12%	8%	13%
1	61%	63%	61%	69%	73%	68%	59%	63%	62%
> 1	10%	10%	10%	15%	15%	14%	28%	29%	24%
Perceived health									
Good/very good	73%	75%	73%	78%	80%	76%	85%	89%	81%
Fair/bad	27%	25%	27%	22%	20%	24%	15%	11%	19%

^a^ Weights were computed based on a regression including age, sex, education, children, country of birth, lifetime same‐sex partners, number of lifetime and 12‐month sexual partners, perceived health, difficulty completing the survey, and mode of survey.

^b^ Weights were computed based on a regression including age, sex, education, financial difficulty, region, country of birth, lifetime same‐sex partners, number of lifetime and 12‐month sexual partners, difficulty completing the phone, and completion of Part 2 survey.

^c^ Weights were computed based on a regression including age, sex, education, country of birth, residence, number of sexual partners in the last 12 months, perceived health, difficulty completing the phone, and completion of Part 2.

#### 4.2.2 Field Worker Feedback

Quantitative participation indicators describe survey performance, but they do not capture how fieldwork unfolded in practice. Interviewer, supervisor, and listener feedback provided additional insight into the operational and relational challenges of administering a large‐scale telephone survey on sexuality.

Overall, interviewers reported that the survey was generally well received. The main operational challenges were handling refusals, applying the household roster procedures when household members were reluctant to identify the eligible respondent, introducing the home self‐sampling kit, and managing some technical aspects of the survey software. The most frequently reported reasons for refusal were lack of time, questionnaire length, and the sensitive nature of the topic. However, field teams indicated that refusals mostly reflected general survey reluctance rather than objection to the subject matter itself. Fear of scam or fraudulent calls was also a recurring barrier during initial contact, underscoring the value of the advance SMS in establishing study legitimacy.

The modules most often described as difficult to administer, and also difficult for respondents to answer, were the sociodemographic block (first and longest), the sexual practices module, and the questions on sexual violence, which could prompt first‐time disclosures. Although discomfort was sometimes apparent, supervisors and listeners reported that respondents generally remained engaged. Field staff also noted that some respondents who were initially hesitant appeared satisfied by the end of the interview. Interviewers highlighted the usefulness of regular exchanges with supervisors and the research team, as well as optional debriefing sessions with a psychologist. Fieldwork nevertheless remained emotionally demanding because of the highly personal and sensitive nature of sexuality and sexual health topics, which sometimes exposed interviewers to respondents' distress and left them feeling intrusive, guilty, or helpless. Interviewers were encouraged to discuss these experiences with the psychologist, either individually or collectively, and could be temporarily reassigned to other projects if they wished to protect their emotional well‐being. They could also discontinue participation in the study at any time without consequences for their employment status.

## 5 Conclusion

This third iteration of the French national survey on sexuality and sexual health (1992, 2006, 2023), conducted in the aftermath of the COVID‐19 pandemic, offers a distinctive opportunity to explore trends in practices, relations, and sexual norms over the past four decades [17]. These decades have witnessed significant transformations, including shifts in gender relations, the proliferation of digital interactions, the emergence of influential social movements like #MeToo, and advancements and challenges in sexual and reproductive health and rights [18].

Consistent with other national population‐based surveys in high‐income settings, response rates have been declining over time, reaching 34% in the current study. This drop is a well‐recognized challenge in survey research, accelerated by the rise of telemarketing, time constraints, survey fatigue, as well as heightened concerns about data privacy. While low, our response rate is well above the average response rate of less than 10% for random digital dial surveys [19]. Additionally, response rates are moderately correlated with non‐response bias [20, 21]. This was made evident in a 2010 French national study on sexual and reproductive health, which used the same methodology as CSF‐2023, and found that increasing response rates by calling back initial refusals increased sample size but also increased sample distortion [22]. We applied post‐stratification weights to address non‐response bias for major sociodemographic characteristics, such as the underrepresentation of younger adults and people with lower educational attainment, with little effect on key SRH indicators, providing some reassurance about sample representativeness. However, unobserved bias may still affect the external validity of the results. For instance, we were unable to assess the representativeness of the sample in terms of racial distribution, as this information is not available in the national census data in France. Likewise, while poststratification accounted for number of household members, we were unable to verify the composition of households, such as single parent households in the poststratification procedures.

Despite this limitation, CSF‐2023 introduces new topics and expands information for established ones, generating previously unavailable data and opening important new avenues for SRH research. In particular, it extends the scope of national survey research on digital sexual health, sexuality at critical life transitions (adolescence, pregnancy, retirement, older ages), and SRH self‐care. The inclusion of STI biomarkers enhances the identification of risk factors and barriers to SRH care across populations, while the large sample also allows an exploration of intersecting social factors informing sexuality and sexual health. CSF‐2023 also addresses critical gaps needed to inform the French National Strategy for Sexual Health [10] and more broadly, Europe's Action plan for sexual and reproductive health: toward achieving the 2030 Agenda for Sustainable Development in Europe [23].

Beyond its substantive contributions, CSF‐2023 also provides valuable methodological insights into the conduct of large‐scale sexual health research. The fieldwork experience underscored the importance of pilot testing to refine concepts, simplify question wording, reduce respondent burden, and improve the sequencing of sensitive modules. It also showed that successful implementation of a sexuality survey depends on strong interviewer preparation and support, including regular supervision and access to psychological support to help field staff manage the emotional demands of discussing intimate and sometimes distressing experiences. These lessons may help guide future population‐based sexual health surveys in France and beyond.

## Funding

This study was supported by Agence Nationale de Recherches sur le Sida et les Hépatites Virales, DOI: 10.13039/501100003323, grant numbers: ECTZ117535. Ministère des Affaires Sociales et de la Santé, DOI: 10.13039/501100004571. Santé Publique France (The French national Public Health Agency). Mission Interministérielle de Lutte Contre les Drogues et les Conduites Addictives, DOI: 10.13039/501100011000. Institut national de la jeunesse et de l'éducation populaire.
